# Aging and CaMKII Alter Intracellular Ca^2+^ Transients and Heart Rhythm in *Drosophila melanogaster*

**DOI:** 10.1371/journal.pone.0101871

**Published:** 2014-07-08

**Authors:** Manuela Santalla, Carlos A. Valverde, Ezequiel Harnichar, Ezequiel Lacunza, Javier Aguilar-Fuentes, Alicia Mattiazzi, Paola Ferrero

**Affiliations:** 1 Centro de Investigaciones Cardiovasculares, CONICET-La Plata, Facultad de Medicina, Universidad Nacional de La Plata, La Plata, Buenos Aires, Argentina; 2 Departamento de Ciencias Básicas y Experimentales, Universidad Nacional del Noroeste de Buenos Aires, Pergamino, Buenos Aires, Argentina; 3 Centro de Investigaciones Inmunológicas Básicas y Aplicadas, CONICET-La Plata, Facultad de Medicina, Universidad Nacional de La Plata La Plata, Buenos Aires, Argentina; 4 Universidad Autónoma de Chiapas, Centro Mesoamericano de Estudios en Salud Pública y Desastres, Nodo Tapachula, Laboratorio de Epigenética del Neurodesarrollo y Neurobiología Molecular, Tapachula, Chiapas, México; Loyola University Chicago, United States of America

## Abstract

Aging is associated to disrupted contractility and rhythmicity, among other cardiovascular alterations. *Drosophila melanogaster* shows a pattern of aging similar to human beings and recapitulates the arrhythmogenic conditions found in the human heart. Moreover, the kinase CaMKII has been characterized as an important regulator of heart function and an arrhythmogenic molecule that participate in Ca^2+^ handling. Using a genetically engineered expressed Ca^2+^ indicator, we report changes in cardiac Ca^2+^ handling at two different ages. Aging prolonged relaxation, reduced spontaneous heart rate (HR) and increased the occurrence of arrhythmias, ectopic beats and asystoles. Alignment between *Drosophila melanogaster* and human CaMKII showed a high degree of conservation and indicates that relevant phosphorylation sites in humans are also present in the fruit fly. Inhibition of CaMKII by KN-93 (CaMKII-specific inhibitor), reduced HR without significant changes in other parameters. By contrast, overexpression of CaMKII increased HR and reduced arrhythmias. Moreover, it increased fluorescence amplitude, maximal rate of rise of fluorescence and reduced time to peak fluorescence. These results suggest that CaMKII in *Drosophila melanogaster* acts directly on heart function and that increasing CaMKII expression levels could be beneficial to improve contractility.

## Introduction

Due to the similarity of genes between flies and humans and the rapid genetic manipulation that offers the fruit fly, *Drosophila melanogaster* has emerged as a model for studies related to cardiac function and dysfunction. Moreover, it is known that *Drosophila melanogaster* has similar proteins responsible for Ca^2+^ handling in the cardiomyocyte, [1] and that adult fruit fly heart recapitulates several aspects of Ca^2+^ regulation observed in human myocardium [2].

Ca^2+^-calmodulin-dependent protein kinase II (CaMKII) is an ubiquitous Ser/Thr-directed protein kinase that is expressed from a family of four closely related genes, α, β, γ and δ whose official symbols are: *CAMK2A*, *CAMK2B*, *CAMK2G* and *CAMK2D*. All CaMKII isoforms in humans and other mammals have numerous splice variants [3]. When intracellular free calcium (Ca^2+^_i_) increases, CaMKII is activated through the binding to the Ca^2+^-calmodulin complex. Once activated, autophosphorylation or oxidation of CaMKII can sustain its activity regardless of the levels of Ca^2+^_i_ [4], [5], and thereby phosphorylate multiple targets [6]–[8]. CaMKII has been associated with cardiac arrhythmias. The arrhythmic pattern triggered by the kinase has been related to the phosphorylation of different CaMKII targets proteins, like phospholamban (PLN), ryanodine receptor (RyR2), L-type Ca^2+^ channel (LTCC) or voltage-gated sodium channels Na(V)1.5 [7], [9]–[12].

*Drosophila melanogaster* CaMKII is codified by a single gene, with several splicing products. This protein also becomes independent of Ca^2+^/calmodulin upon autophosphorylation [13]. Given the crucial role of CaMKII in Ca^2+^ handling, excitability, contractility and cardiac pathology in humans, and being that *Drosophila melanogaster* constitutes a good model organism, it is of great importance to assess the role of this kinase in the fruit fly heart. However, a possible participation of CaMKII in *Drosophila melanogaster* heart regulation has never been considered.

In the present study we used a *Drosophila melanogaster* semi-intact heart model that express the Ca^2+^ reporter system GCaMP3 (a genetically encoded calcium indicator), with two main goals: 1. To characterize the intracellular Ca^2+^ behavior of *Drosophila melanogaster* heart in young (7 days) and old (60 days) flies; 2. To investigate a possible role of CaMKII in the regulation of cardiac function in young adult flies.

It will be shown that the fly model expressing GCaMP3 is a convenient experimental tool to assess intracellular Ca^2+^ dynamics in young and senescent *Drosophila melanogaster* hearts. Moreover, CaMKII appears to play a significant role in *Drosophila melanogaster* heart function regulation and deregulation.

## Results

### 1. Characterization of the model of *Drosophila melanogaster* heart expressing the genetically encoded Ca^2+^ indicator at two different ages

We used transgenic flies harboring UAS-associated GCaMP3 construct and the GAL4 transcriptional activator protein, under the control of the cardiac-specific tinC driver. GCaMP3 is a green fluorescent protein that, like GCAMP2, includes a Ca^2+^/calmodulin binding domain and a peptide from myosin light chain kinase, M13. According to what has been described, the genetic indicator GCaMP3 is brighter, has greater protein stability and a larger dynamic range and higher affinity for Ca^2+^ compared to GCaMP2 [14], [15]. When Gal4 binds to UAS sequence, induces production of GaMP3. Since maximal activity of Gal4 occurs between 28 and 29°C, GCaMP3 expression can be induced 48 hours before the selection of the flies for dissection, avoiding the permanent expression of this protein in heart tissue.

**Figure 1:**
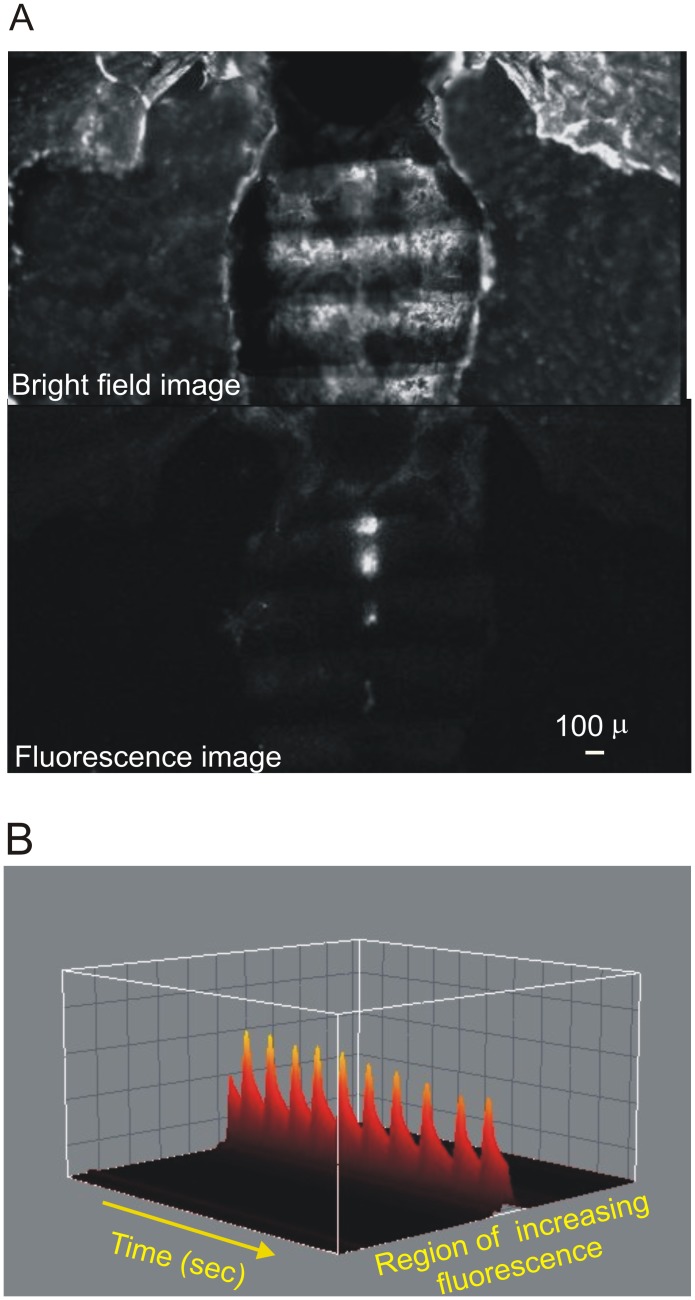
Visualization of fluorescence Ca^2+^ signal in semi intact preparations. A. Top: Bright-field image of a fruit fly with heart exposed after removal of abdominal organs. Bottom: fluorescent signal of GCaMP3 protein that senses local increasing of Ca^2+^ concentration. B. 3D representation of fluorescent peak recordings on the conical chamber–first abdominal segment - along time.

Using fluorescence microscopy we observed semi-intact heart preparations from 7 and 60 days old flies maintained in oxygenated supplemented artificial hemolymph. **Figure 1A** shows representative bright-field image (top) and fluorescence image (bottom) of a heart prepared from a 7 days fly expressing the fluorescent Ca^2+^-sensing protein, GCaMP3. Typical recordings of Ca^2+^ transients are also shown in panel B of **Figure 1**.

**Figure 2:**
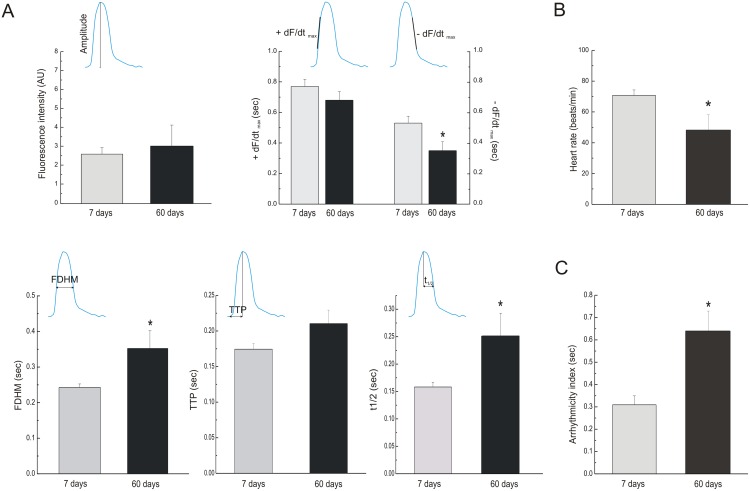
Effects of aging on functional parameters measured through the genetic codified indicator. A. Old flies did not exhibit changes of fluorescence intensity (Top and left graph) neither +dF/dt_max_ compared with younger ones, but reduction of –dF/dt_max_ was observed with aging (Top and right graph). Old flies exhibited prolongation of FDHM (Bottom and left graph) without significantly changes in TTP duration (Bottom and middle graph) but they shown a lengthening of half relaxation time relative to young flies (Bottom and right graph). B. Old flies presented reduction of spontaneous heart rate. C. An increase of arrhythmicity index was observed in 60 days old flies compared with 7 days old individuals. Mean ± SEM of 57 flies. *p≤0.05.

**Figure 3:**
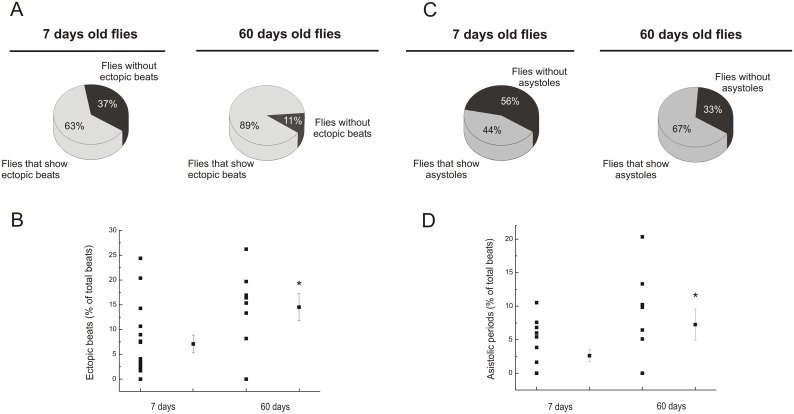
The incidence of ectopic beats and asystoles increases with aging. A. The number of individuals that exhibited ectopic beats was greater in old flies, compared with younger ones. B. The percentage of ectopic beats over total beats was increased in old flies. C. The number of individuals that exhibited asystoles was greater in old flies, compared with younger ones. D. The percentage of asystoles as function of total beats was increased in old flies. Mean ± SEM of 57 flies. *p≤0.05.

Different cardiac functional changes during aging have already been described in *Drosophila melanogaster* [16]–[21]. These studies used different approaches, e.g. optical coherence tomography and direct visualization of chamber dimensions changes, without direct measurements of Ca^2+^ dynamics. Using the genetic approach developed by Lin *et al*. [14], we characterized changes related to Ca^2+^ dynamics with aging. **Figure 2** compares overall results of different characteristics of Ca^2+^ transients obtained in heart flies at 7 and 60 days. As can be appreciated, senescence did not modify Ca^2+^ transient amplitude and maximal rate of rise of fluorescence (+dF/dt_max_), meanwhile, maximal rate of fluorescence decay (–dF/dt_max_) was significantly diminished. This finding is consistent with a prolongation of full duration at half maximum (FDHM) of Ca^2+^ fluorescence produced by a significant increase in half relaxation time. Taken together, these findings indicated that senescence produces a significant decline in the relaxation process. Moreover, senescence decreased spontaneous frequency and increased what has been called arrhythmicity index [16], which actually reflects the variability in heart frequency, in agreement with previously reported findings [16]. In addition, we counted ectopic beats -defined as events that occur either during the relaxation phase of a given beat or immediately after- and asystoles -defined as periods with duration longer than two standard deviations above the average period. The incidence of this type of irregular events (ectopic beats and asystoles), was significantly greater in old flies than in young individuals (**Figure 3, A and C**). Moreover, the number of events, expressed as percentage of total beats, was significantly greater in 60 days old than in young flies (**Figure 3, B and D**). Clearly, the highly rhythmic beating pattern observed in young flies deteriorates with age, and by 60 days, most of WT flies exhibits non-rhythmical heart contraction patterns, including asystoles/bradycardia and ectopic beats. Consequently, the results validate the use of the genetically expressed indicator, GCaMP3, to assess Ca^2+^ handling in the fruit fly heart and indicate that major changes produced by senescence are related to a decrease in spontaneous frequency, the increased incidence of arrhythmogenic events and the slowing of intracellular Ca^2+^ decay. Interestingly, these results recapitulate typical characteristics of mammalian senescent myocardium [22], [23].

### 2. CaMKII from *Drosophila melanogaster* is highly conserved in the human homologs

With the aim of determining the human homolog of the *Drosophila melanogaster* CaMKII, a BLAST search alignment using the HomoloGene tool was performed. Interestingly, *CAMK2D* variant, which is present in heart tissue, has the highest homology with the *Drosophila melanogaster* CaMKII. Furthermore, in comparison with other mammalian species, human CaMKII has one of the highest scores of similarity to fruit fly ortholog [**Table S1**]. We then determined the degree of similarity between the different isoforms of CaMKII from *Drosophila melanogaster* and the isoforms of the human CaMKIIδ. To this purpose, all splice variants of CaMKIIδ from human and CAMKII from fruit fly were obtained and aligned. Scores ranged between 74 and 81%. All *Drosophila melanogaster* isoforms showed the highest percentage of similarity with the isoforms 1 and 2 of the human *CaMK2D*, with a slightly predominance of the isoform 2 (**Table 1**).

**Table 1:** CaMKII from *Drosophila melanogaster* exhibit high homology with the product of human gene CaMK2D.

CAMKII(*D melanogaster*)	CAMKH2D(*H sapiens*)	Score	Identities (%)	Positives	Gaps
Isoform A	Isoforms 2, 1	825	81	432/478(90%)	3/478(0%)
Isoform B	Isoforms 2, 1	817	78	432/497(86%)	22/497(4%)
Isoform C	Isoforms 2, 1	825	81	432/478(90%)	3/478(0%)
Isoform D	Isoforms 2, 1	810	75	432/518(83%)	43/518(8%)
Isoform E	Isoforms 2, 1	817	78	432/497(86%)	22/497(4%
Isoform G	Isoforms 2, 1	816	77	432/504(85%)	29/504(5%)
Isoforma H	Isoforms 2, 1	825	81	432/478(90%)	3/478(0%)
Isoform I	Isoforms 2, 1	810	75	432/518(83%)	43/518(8%)
Isoform J	Isoforms 2, 1	809	74	432/519(83%)	44/519(8%)
Isoform K	Isoforms 2, 1	820	81	429/477(89%)	9/477(1%)
Isoform L	Isoforms 1, 2	801	74	429/517(82%)	49/517(9%)
Isoform M	Isoform 2	807	77	429/499(85%)	31/499(6%)

All isoforms of *Drosophila melanogaster* CaMKII were aligned with the human genome and we found that CaMKII of the fruit fly has high homology with the product of gene *CaMK2D*, the most important human cardiac variant. The table shows that all splice variants from *Drosophila melanogaster* possess high scores with splice variants 1 and 2 of *CaMK2D* gene.

**Figure 4:**
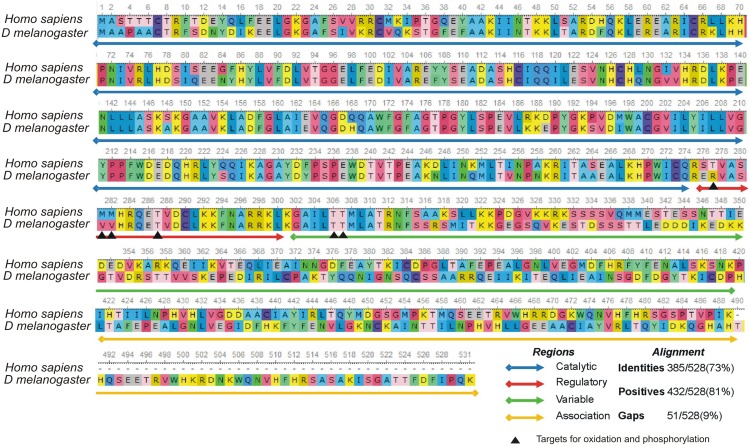
CaMKII from human and *Drosophila melanogaster* are highly conserved. Alignment between CaMKIIδ from *Homo sapiens* and the corresponding enzyme in *Drosophila melanogaster* show conserved regions and residues of interest that are targets of phosphorylation. Thr^287^, Thr^306^ and Thr^307^ on human isoform have their homologous residues in *Drosophila melanogaster*. This might allow *Drosophila melanogaster* protein to be regulated by autophosphorylation like occurs in its human homologous enzyme. Unlike phosphorylation sites, Met^281/282^ oxidation sites are replaced by valine residues in the fruit fly, thus, regulation by oxidation of Met^281/282^, are absent in *Drosophila melanogaster*.

**Figure 4** shows the alignment between human CaMKIIδ and *Drosophila melanogaster* CaMKII. Catalytic, regulatory, variable and association domains are highlighted. Arrows indicate sites of functional interest like phosphorylation and oxidation residues.

The regulatory domain of human CaMKIIδ includes features of calmodulin binding and autophosphorylation through the threonine^287^, which upon phosphorylation allows the enzyme to persist active, independently of intracellular Ca^2+^ levels. Phosphorylation of Thr^306^/Thr^307^ is mediated by Thr^287^-phosphorylated CaMKII. This phosphorylation (Thr^306/307^) is relevant to regulate the auto-activated form of the enzyme [24]. It has been described that neuronal CaMKII from *Drosophila melanogaster* has similar phosphorylation sites to that of the neural CaMKIIα from rat [25]. Moreover, human CaMKIIδ possess two important sites- Met^281^/Met^282^, capable to be oxidized by reactive oxygen species (ROS). Activation of CaMKII by ROS evokes, similar to autophosphorylation, Ca^2+^ autonomous activity [26]. Figure 4 shows that the phosphorylation sites are conserved between human and *Drosophila melanogaster* CaMKII. Instead, methionines are replaced by valine residues in the fruit fly.

Taken together the above results indicate a high degree of similarity between human and *Drosophila melanogaster*. Moreover, the fly fruit conserves fundamental residues for CaMKII function. This prompted us to continue with functional analysis of this protein in the fruit fly heart.

### 3. CaMKII inhibition reduces spontaneous frequency but does not affect basal Ca^2+^ transient amplitude and Ca^2+^ transient decay

**Figure 5:**
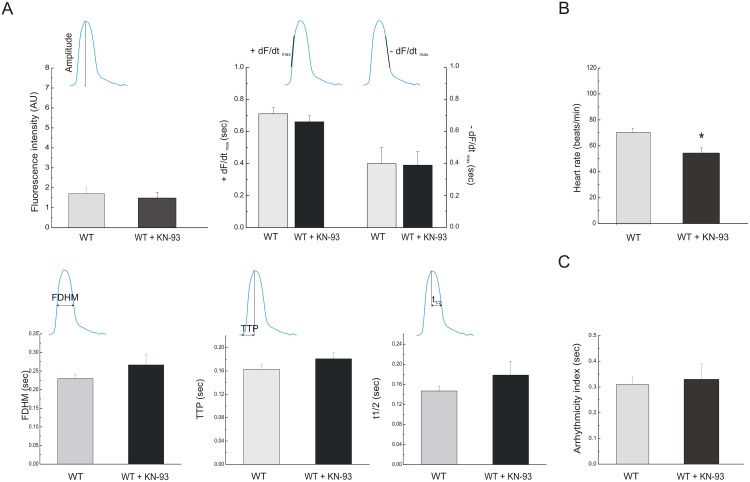
Inhibition of CaMKII with KN-93 affects the spontaneous frequency of the heart. A. Flies of 7 days treated with 5 µM KN-93 did not exhibit changes of fluorescence intensity (top, left graph), +dF/dt_max_, −dF/dt_max_ (top, right graph), FDHM (bottom, left graph), TTP (bottom, middle graph) neither t_1/2_ (bottom, right graph) compared whit the same 7 days flies previously observed without incubated with the CaMKII inhibitor. B. Inhibition of CaMKII reduced spontaneous heart rate without changes of the arrhythmicity index. Mean ± SEM of 24 flies. *p≤0.05.

As already stated, CaMKII regulates many of the key proteins that are involved in the excitation-contraction coupling (ECC) of human heart. There is an increasing awareness that disorders of ECC are at the basis of different type of arrhythmias and may conduct to cardiac hypertrophy and heart failure. Thus, knowledge of the effect of CaMKII in a model like *Drosophila melanogaster* heart may provide a useful tool to gain insights into the mechanisms of cardiac failure and arrhythmias in human myocardium. We therefore explored the effects of CaMKII inhibition in *Drosophila melanogaster* heart. **Figure 5A** shows that inhibition of CaMKII by 5 µmol/L of the specific CaMKII inhibitor, KN-93, failed to affect the different parameters used to characterize contractility and relaxation. On the other hand, it produced a significant decrease in spontaneous frequency (**Figure 5B**) without affecting the arrhythmicity index (**Figure 5C**). These results are consistent with previous findings in mice lacking a major myocardial CaMKII isoform (CaMKIIδ) [27], [28] and mice with myocardial expression of a CaMKII inhibitory peptide exhibiting normal baseline ventricular function and ECC parameters [29], which in turn have a reduced heart rate response to catecholamine stimulation by actions at sinoatrial nodal pacemaker in mammalian cells [30].

**Figure 6:**
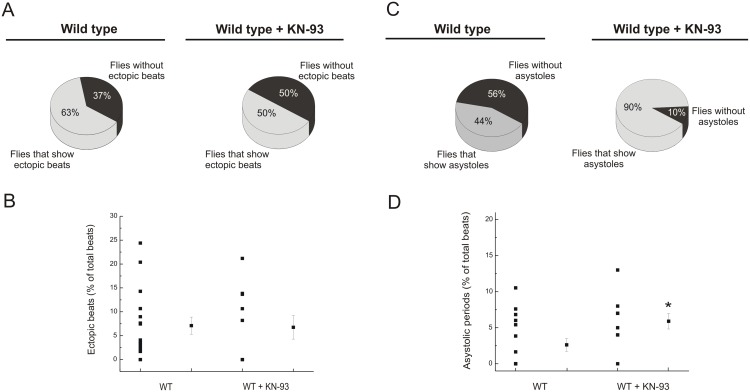
CaMKII inhibition does not modify the occurrence of ectopic beats but increased the incidence of asystoles. Inhibition of CaMKII did not change significantly the number of individuals that exhibited ectopic beats (A) neither the percentage of ectopics beats over total beats (B). The number of flies that showed asystoles was greater with CaMKII inhibition (C). The percentage of asystoles in these flies referred to the total beats, was significantly different from observations without KN-93 (D). Mean ± SEM of 24 flies. *p≤0.05.

The incidence of flies that showed ectopic beats with CaMKII inhibition was slightly lower than that observed without KN-93, while ectopic beats, expressed as percentage of total beats, was similar in both groups (**Figure 6, A and B**). Instead, the fraction of flies that exhibited asystoles was increased with CaMKII inhibition and these events were more frequent than in control flies (**Figure 6, C and D**).

Taken together the results showed that CaMKII inhibition decreased spontaneous heart rate and increased the incidence of asystoles.

### 4. CaMKII overexpression increased spontaneous frequency, decreased frequency variability and modified intracellular Ca^2+^ dynamics

**Figure 7:**
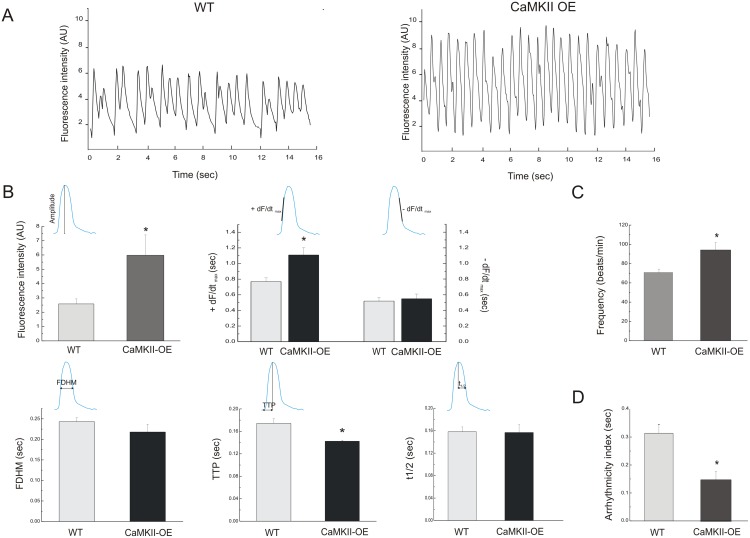
Overexpression of CaMKII increases heart rate, reduces arrhythmogenesis and improves Ca^2+^ dynamics. A: Typical records of control flies and heterozygous flies that contain one extra copy of CaMKII (CaMKII-OE). Flies that overexpress the kinase exhibited increased spontaneous frequency and fluorescence amplitude compared with control flies. B: Summary data showed changes in transient Ca^2+^ dynamics, like the rise of fluorescence amplitude (top, left graph) and the maximal rate of rise of fluorescence +dF/dt_max_ without changes in −dF/dt_max_ (top, right graph). FDHM was not changed with CaMKII overexpression (bottom, left graph) and TTP was shortened (bottom, middle graph) without changes of t_1/2_ (bottom, right graph). An increment of spontaneous frequency (C) and reduction of arrhythmicity index (D) was observed in CaMKII-OE flies. Mean ± SEM of 28 flies. *p≤0.05.

We carried out experiments with heterozygous strains that contain one extra copy of CaMKII gene and transcript expression was corroborated by RT-PCR (**SI,** **Fig. 2**). In an additional set of experiments, we proceeded to the functional analysis. **Figure 7A** depicts typical records of intracellular Ca^2+^ transient of wild type control flies (left panel) and individuals that overexpress CaMKII (CaMKII-OE, right panel). Cardiac CaMKII overexpression increased the amplitude of intracellular Ca^2+^ transients and spontaneous heart rate. The increase of amplitude in CaMKII-OE flies was associated with a significant enhancement in +dF/dt_max_ and a shortening of TTP of Ca^2+^ transient (**Figure 7B**). Besides the increment of spontaneous heart rate (**Figure 7C**), a reduction of the arrhythmogenic index was observed in CaMKII-OE flies (**Figure 7D**). These results indicate that CaMKII has a main role in ECC regulation in *Drosophila melanogaster* heart.

**Figure 8:**
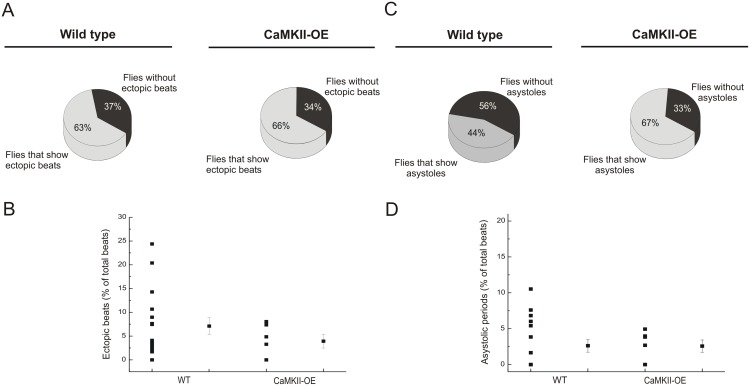
CaMKII overexpression did not affect the occurrence of ectopic beats and asystoles. CaMKII-OE flies did not exhibit changes on ectopic beats and asystoles compared with WT flies (A, C). Percentage of ectopic beats and asystoles over total beats were not different between control and CaMKII-OE flies (B, D). Mean ± SEM of 28 flies. *p≤0.05.

Incidence of ectopic beats in CaMKII-OE flies was similar to the incidence of events observed in the control group. Moreover, CaMKII-OE individuals showed no significant changes in the number of atypical events over total beats. This result is consistent with a reduction of the arrhythmicity index (**Figure 8, A and C**). Moreover, a greater fraction of CaMKII-OE flies showed asystoles, although the percentage of long periods over total beats was similar between control flies and CaMKII-OE (**Figure 8, B and D**).

## Discussion

In the present study, we used an alternative animal model to the commonly explored mammals for cardiovascular studies -the fruit fly *Drosophila melanogaster*-, taking advantage of its easy manipulation. Previous cardiac reports *in Drosophila* highlighted the conservation of developmental genes in the fruit fly relative to mammals [31], [32]. Pioneer works mainly from Bodmer and Rockman groups, clearly revealed the importance of *Drosophila melanogaster* heart research to provide new insights into human heart function [2], [19]. Later, pharmacological and genetic approaches showed that heart rate in larval and adult flies is regulated by similar proteins to those that control ECC in humans [1], [33]. Additional studies were focused on aging [16], [17], [20]. For instance, experiments from Wolff’s laboratory recently demonstrated that *Drosophila melanogaster* hearts have readily measureable Ca^2+^ fluorescent signals that are dependent on L-type Ca^2+^ channels and SR Ca^2+^ stores. These authors used engineered transgenic *Drosophila melanogaster* that have cardiac-specific expression of the fluorescent Ca^2+^-sensing protein, GCaMP2 [14]. In the present experiments we took advantage of this model to characterize intracellular Ca^2+^ transients at two different ages: 7 and 60 days of life.

In all these approaches, morphological assessment of cardiac features by optical coherence tomography (OCT) and shortening of heart walls by recording of high speed videos, provided information about functional parameters [2], [16], [20]. More recently, Ca^2+^ handling could be measured by genetically codified indicators [14].

In the present report, we used the transgenic flies that harbor genetic codified calcium indicator [14], focusing on changes in Ca^2+^ handling with aging and the possible role of the multifunctional CaMKII on cardiac function.

Our results showed for the first time that 1. Aging alters intracellular cardiac Ca^2+^ dynamics, prolonging the decay of Ca^2+^ transient in *Drosophila melanogaster*. 2. Aging increases the incidence of ectopic beats and asystoles. 3. CaMKII gene from *Drosophila melanogaster* is homolog to mammalian *CaMK2D* gene and CaMKII from the fruit fly conserves important fundamental phosphorylation residues for CaMKII function. 4. CaMKII from *Drosophila* regulates spontaneous frequency, frequency variability and intracellular Ca^2+^ dynamics.

These findings emphasize the importance of *Drosophila melanogaster* cardiac preparation as a useful tool to gain important insights into human cardiac health and disease.

### Cardiac aging in *Drosophila melanogaster*

An important finding of this study is that aging modified intracellular Ca^2+^ dynamics by slowing cardiac relaxation. Similar findings have been described in humans, whose myocardial contraction is also prolonged and relaxation is incomplete in aged individuals compared with younger adults [23].

The present results also showed that old flies exhibited a reduction in spontaneous cardiac frequency than younger flies. Moreover frequency variability was increased in the older flies, in agreement with previous reports by Ocorr *et al*., using OCT and high speed movies of movement of heart wall [16].

Importantly, our results also demonstrated an increase in ectopic beats and asystoles with aging. The study carried out by Ocorr *et al*. in this model [16], showed an association between heart dysfunction and increased arrhythmogenic index with a reduction in the expression of the *Drosophila* homolog of human KCNQ1- encoded K^+^ channel α subunits. Whether the predominance of ectopic beats described here can also be ascribed to these channels is not apparent from the present findings.

### CaMKII regulates cardiac function in *Drosophila melanogaster* heart

CaMKII can phosphorylate different key proteins of the ECC, like RyR, PLN, the Na(V)1.5 channels and LTCC. Phosphorylation of these proteins has been involved in the pathogenesis of different type of arrhythmias in mammals [9], [12], [34]–[36].

In the present study, we showed a high degree of similarity between *Drosophila* CaMKII and the human product of gene *CaMK2D*, the most predominant isoform in heart tissue. Previous reports about similarities of CaMKII in *Drosophila* and mammals have referred to its presence and functionality in brain [37], [38]. More recently, a CaMKII isoform has been detected in *Drosophila* heart through mass spectrometry [39]. However, a possible role of CaMKII in *Drosophila* heart was not identified.

In the present report, we found that inhibition of CaMKII in young adults did not significantly affect Ca^2+^ dynamics. These results are in agreement with studies in mice hearts in which either the absence of CaMKIIδ, a major myocardial CaMKII isoform, or the cardiac expression of a CaMKII inhibitory peptide, does not alter ECC under basal conditions [27]–[29], and with a recent report showing that cardiac knock down of CaMKII alone in *Drosophila melanogaster* did not affect heart function [40]. In contrast, the present findings demonstrated that CaMKII overexpression increased Ca^2+^ transient amplitude and reduced time to peak of Ca^2+^ transient amplitude, a result that demonstrated a direct role of CaMKII on heart function in *Drosophila melanogaster* adult heart. These results are at odds with the results obtained with chronic overexpression of CaMKII in mouse hearts. In these mammals, overexpression of CaMKII produced changes in ECC, like a decrease in Ca^2+^ transient amplitude and SR Ca^2+^ content, which predispose these animals to the progression to heart failure stage [41]. The cause for the different action of CaMKII in *Drosophila* vs. mammalian heart function is not apparent from the present results. Whether the different circulatory systems: open (*Drosophila*) vs. closed (mammals) can influence the dissimilar impact of CaMKII in *Drosophila* and mammalian heart is difficult to ascertain at present. Possible other reasons for this difference may be searched on putative different phosphorylations targets for CaMKII in both species, different regulation of CaMKII activity (in mammals, CaMKII activity is regulated by autophosphorylation and oxidation, whereas the fruit fly conserves phosphorylation sites but lacks oxidation sites), or different long term effects of the overexpression of the kinase through the activation of different transcription factors. Future work need to be performed along these lines to definitively understand this phenomenon.

Moreover, the present results showed that CaMKII inhibition reduced spontaneous frequency and increased the fraction of asystoles. Moreover, CaMKII overexpression increased spontaneous frequency and reduced frequency variability (arrhythmicity index of Ocorr, 2007) with little effect on ectopic events.

The underlying mechanism of CaMKII role on spontaneous frequency and arrhythmias remains to be elucidated. It has been reported in rabbit heart, that spontaneous rhythmic excitation of sinoatrial (SA) node cells is critically dependent on CaMKII, which is highly localized beneath the surface membrane in the vicinity of L-type channels and modulates L-type Ca^2+^ current (ICa) [42]. Our recording of heart activity is localized on the conical chamber, a putative pacemaker that leads the retrograde displacement of hemolymph [43]. CaMKII might regulate pacemaker activity of conical chamber in addition to phosphorylate LTCC.

Taken together, the results indicate that CaMKII modifies different properties of *Drosophila* heart, *i.e.*, inotropism and lusitropism, because it increases the amplitude of intracellular Ca^2+^ transients and the speed relaxation and chronotropism, because it regulates spontaneous frequency and heart rate variability. Ongoing experiments in our laboratory are studying the possible influence of CaMKII activation in aging, with the goal of providing further insights in this possible and still unknown relationship.

In summary, the results indicate that CaMKII exerts a beneficial effect by improving cardiac performance and reducing heart rate variability. Although CaMKII is described in mammalian heart as an arrhythmogenic molecule, we did not find evidence that this was also the case for *Drosophila* heart. Future exhaustive analysis will permit to explore possible compensatory mechanisms of chronic overexpression of CaMKII in *Drosophila melanogaster.*

## Materials and Methods

### Homology analysis of the CAMKII sequences from human and *Drosophila melanogaster*

Sequences were obtained from the NCBI Gene database (http://www.ncbi.nlm.nih.gov/pubmed/Gene), uploaded to UGENE program (Unipro UGENE, Version 1.12.2), and the multiple sequence alignment was performed using integrated CLUSTAL and Kalign algorithms.

Pairwise alignment scores of the different species were obtained using the HomoloGene tool of the NCBI (http://www.ncbi.nlm.nih.gov/homologen).

### Stocks amplification and genetic crosses

*Drosophila melanogaster* stocks were maintained and amplified at 28°C on standard cornmeal-yeast medium. Flies of strain *UASGCAMP3 TinC Gal4, UASGCAMP3* were generously provided by Dr Mathew Wolf (Duke University School of Medicine, USA). These transgenic flies express the reporter system GCaMP3 downstream the UAS sequence, and the Gal4 protein under control of TinC promoter, specifically expressed in the heart. 7 and 60 days old individuals were selected for experiments.

We also amplified flies with the genotype w[*]; P{w[+mC] = UAS-CaMKII.R3}2 that contains the CaMKII gene downstream UAS sequence. This strain was obtained from Bloomington Stock Center (Indiana, USA). We collected virgin females to cross with the stock that contains the reporter system. Offspring selectively expresses CaMKII in the heart, under the control of *TinCGal4*. Individuals heterozygous of 7 days were collected to obtain semi-intact preparations.

### Heart dissection

Flies collected at 7 and 60 days after eclosion were used for cardiac Ca^2+^ fluorescence measurements. Hearts were prepared according to previously described methods [44]. Briefly, adult flies were anesthetized with CO_2_ and placed on a soft gel plate dorsal side down. Thorax and legs were removed from the abdomen using iridectomy scissors. In this way, we studied the myogenic contraction without influences from neuronal input, as described in previous reports [16]. Flies were bathed in oxygenated hemolymph buffer (in mM): NaCl, 108; KCl, 5; MgCl_2_, 8; NaH_2_PO_4_, 1; NaHCO_3_, 4; HEPES, 5 (pH 7.1); sucrose, 10; Trehalose, 5; and CaCl_2_, 2, at room temperature. The ventral abdominal cuticle was removed and abdominal organs were gently dissected away from the heart. Surrounding fat was removed by liposuction using glass capillaries. The heart remained attached to the dorsal cuticle and was readily identified as a beating structure along the midline from abdominal segments A1 to A4. We registered the signal on the conical chamber, positioned on the first abdominal segment, where the fluorescence intensity was higher than in other regions of the preparation.

### Functional analysis

Dissected hearts were observed using a confocal microscope (Carls Zeiss 410). We scanned changes in fluorescence of the conical chamber, localized in the first abdominal segment. Each fluorescence increase followed by a fluorescence decrease represents the transient elevation of cytosolic Ca^2+^ that precedes contraction. Recordings of Ca^2+^ transients were carried out for as long as 26 seconds. Images were analyzed with ImageJ (National Institutes of Health, USA) and LabChart software (AD Instruments, CO, USA).

Experiments were performed on individuals carrying the reporter system only, which we called control or wild type (WT) and individual heterozygous carrying one additional copy of CaMKII (CaMKII-OE). In a group of experiments, we used 5 µMol/L of KN-93 (Calbiochem, USA) to inhibit CaMKII in WT flies.

The following parameters were analyzed from Ca^2+^ transients: peak Ca^2+^ transient amplitude, expressed in arbitrary units of fluorescence intensity, maximal rate of rise of fluorescence (+dF/dt_max_), maximal rate of fluorescence decay (−dF/dt_max_), full duration at half maximum (FDHM), time to peak (TTP) and half-time of peak fluorescence decline (t_1/2_). We measured heart period, as the interval between two consecutive peaks of maximal fluorescence, to analyze irregularities in heart rhythm, i.e. frequency variability, through the arrhythmicity index according Ocorr *et al.* [16].

A summary of experimental procedures and parameters described is shown by Fig. S1.

### Analysis of transcripts expression

Hearts of WT and CaMKII-OE flies were removed, collected and maintained in RNA later (Quiagen Inc). RNA extraction was carried out with RNAeasy kit, according to the manufacturer’s instructions (Quiagen Inc). RT-PCR was performed using SuperScript reverse transcriptase (Quiagen Inc). PCR reaction conditions were as follows: step 1: 95°C for 30 seconds, Step 2: 40 cycles of 95°C for 30 sec followed by 55°C for 1 min and 72°C for 30 sec.

Primers to identify a 101 pairs of bases fragment of CaMKII cDNA were designed with the following sequences:

Forward primer: ACAACCATTTTAAATCCTCATGTGC

Reverse primer: TGTGCATGTCCTTGTTATCA

Tubulin α-1 was used as control and primers to identify a 182 pairs of bases fragment of the cDNA were designed with the following sequences:

Forward primer: ATCAACTACCAGCCTCCCAC

Reverse primer: TCCTCCATCCCCTCCCCAAC

Samples containing 40 ng of RNA were separated by electrophoresis on 2% agarose gel and visualized with SYBR Safe DNA Gel Stain (Invitrogen, USA).

### Statistics

All results are expressed as mean ± SEM. Comparisons were made using Student’s unpaired *t* test. Values of P≤0.05 were considered statistically significant. Due to the exploratory nature of the present study, multiple, unrelated hypothesis were tested using a single control group.

## Supporting Information

Figure S1 **Summary of experimental procedures to obtain semi-intact preparation and recording of fluorescent signal.** A: Intact fly anesthetized, positioned with dorsal side down, on the Petri dish. B: Head, thorax and abdominal organs were removed. C: abdominal region with beating heart attached to cuticle through alary muscles was exposed. Preparation was bathed with artificial oxygenated hemolymph. Heart was visualized by bright field microscopy and then, fluorescent image was obtained by excitation of preparation with laser 488. Emission was recording and transformed in digital signal to be analyzed. D. Parameters measured are indicated.
(TIF)

Figure S2 **Detection of transcripts for CaMKII in heart tissue from control and CaMKII-OE flies.** Total RNA was extracted from 120 isolated hearts from control and CaMKII-OE flies. CaMKII was amplified and visualized by electrophoresis on gel of agarose 2%. The observed amplified product of 101 base pairs (bp) correspond to CaMKII detected in brain and hear tissue form control and heterozygous with one extra copy of gene. Fragment of alpha-tubulin of 182 bp was utilized as control.
(TIF)

Table S1 **Pairwise alignment scores between CaMKII from** ***Drosophila melanogaster*** **against different mammalian species.** Official gene symbol for each species is indicated. *Drosophila melanogaster* and *Homo sapiens* have a greater percentage of identity compare with other common mammalian species used as model for cardiac studies.
(DOC)
