# Supplementary figures and images for: Aging and CaMKII Alter Intracellular Ca2+ Transients and Heart Rhythm in Drosophila melanogaster
Source: PLoS One. 2014 Jul 8;9(7):e101871. doi: 10.1371/journal.pone.0101871 (PMC4087024; doi:10.1371/journal.pone.0101871)

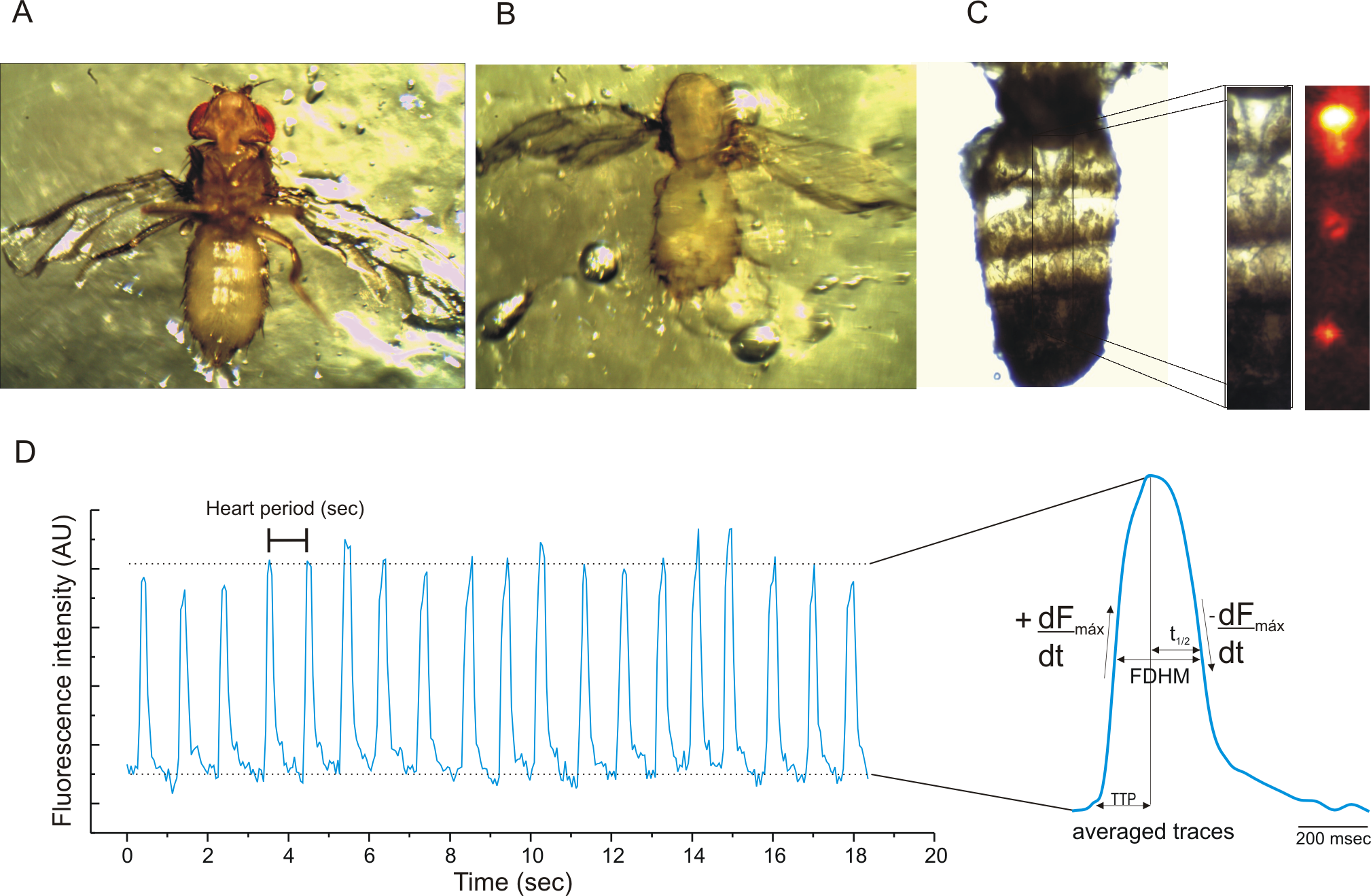

Supplement: Figure S1 — Summary of experimental procedures to obtain semi-intact preparation and recording of fluorescent signal. A: Intact fly anesthetized, positioned with dorsal side down, on the Petri dish. B: Head, thorax and abdominal organs were removed. C: abdominal region with beating heart attached to cuticle through alary muscles was exposed. Preparation was bathed with artificial oxygenated hemolymph. Heart was visualized by bright field microscopy and then, fluorescent image was obtained by excitation of preparation with laser 488. Emission was recording and transformed in digital signal to be analyzed. D. Parameters measured are indicated. (TIF) [file pone.0101871.s001.tif]

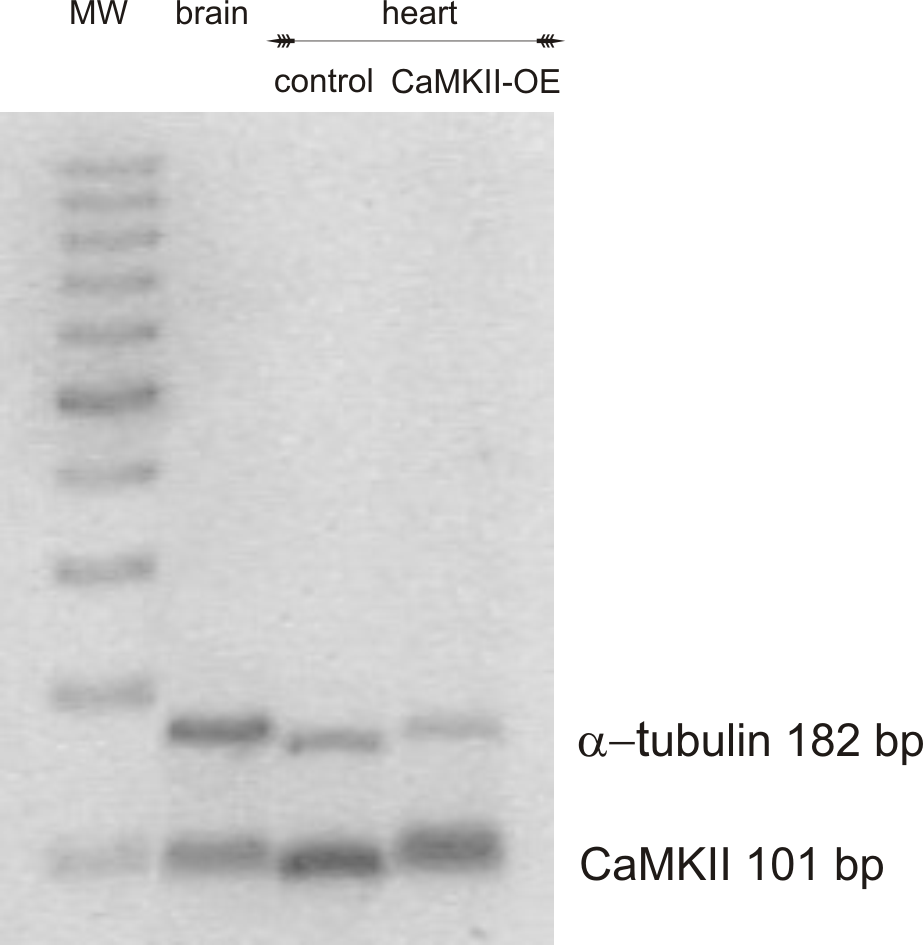

Supplement: Figure S2 — Detection of transcripts for CaMKII in heart tissue from control and CaMKII-OE flies. Total RNA was extracted from 120 isolated hearts from control and CaMKII-OE flies. CaMKII was amplified and visualized by electrophoresis on gel of agarose 2%. The observed amplified product of 101 base pairs (bp) correspond to CaMKII detected in brain and hear tissue form control and heterozygous with one extra copy of gene. Fragment of alpha-tubulin of 182 bp was utilized as control. (TIF) [file pone.0101871.s002.tif]
