# Supplementary material for: Aging and CaMKII Alter Intracellular Ca2+ Transients and Heart Rhythm in Drosophila melanogaster
Source: PLoS One. 2014 Jul 8;9(7):e101871. doi: 10.1371/journal.pone.0101871 (PMC4087024; doi:10.1371/journal.pone.0101871)
Supplement: Table S1 — Pairwise alignment scores between CaMKII from Drosophila melanogaster against different mammalian species. Official gene symbol for each species is indicated. Drosophila melanogaster and Homo sapiens have a greater percentage of identity compare with other common mammalian species used as model for cardiac studies. (DOC) [file pone.0101871.s003.doc]

**Table S1.** Pairwise alignment scores between CaMKII from *Drosophila melanogaster* against different mammalian species.

| **Species** | **Gene symbol** | **Protein Identity (%)** |
| --- | --- | --- |
| *D melanogaster* | CaMKII |  |
| *Canis lupus* | CAMK2D | 80.9 |
| *Homo sapiens* | CAMK2D | 80.7 |
| *Pan troglodytes* | CAMK2D | 80.7 |
| *Bos taurus* | CAMK2D | 80.5 |
| *Macaca mulatta* | CAMK2D | 74.9 |
| *Mus musculus* | Camk2d | 74.9 |
| *Rattus norvegicus* | Camk2d | 74.9 |
